# Adult T-Cell Leukemia-Lymphoma during Pregnancy

**DOI:** 10.1155/2013/631825

**Published:** 2013-06-13

**Authors:** Martin Miguel Amor, Aedan Simon Olaso, Robert Atienza, Benjamin Stueben, Seth Cohen, Plamen Kossev

**Affiliations:** ^1^Department of Internal Medicine, Monmouth Medical Center, Long Branch, NJ 07740, USA; ^2^Department of Pathology, Monmouth Medical Center, Long Branch, NJ 07740, USA; ^3^Section of Hematology/Oncology, Monmouth Medical Center, Long Branch, NJ 07740, USA

## Abstract

Adult T-cell leukemia-lymphoma (ATL) is an uncommon highly aggressive T-cell lymphoma associated with human T-cell lymphotropic virus type 1 (HTLV-1) infection. It is rarely encountered during pregnancy and is particularly challenging to treat due to its aggressive nature and because of the lack of robust data on optimal chemotherapy. We report a case of a Jamaican immigrant diagnosed with ATL during pregnancy.

## 1. Introduction

Adult T-cell leukemia-lymphoma (ATL) is an uncommon lymphoid malignancy associated with human T-cell lymphotropic virus type 1 (HTLV-1) infection. HTLV-1 infection is endemic in Japan and Caribbean basin and occurs sporadically in Africa, Central and South America, Middle East, and southeastern United States. The global seroprevalence of HTLV-1 is 4.4% [1], with higher prevalence in endemic areas. ATL develops in 2%–4% of HTLV-1 infected people [2]. There were only 3 previously reported cases of ATL during pregnancy based on the reporters' comprehensive review of the literature [3–5]. We report a case of a Jamaican female diagnosed with ATL during pregnancy.

## 2. Case Report

**Figure 1:**
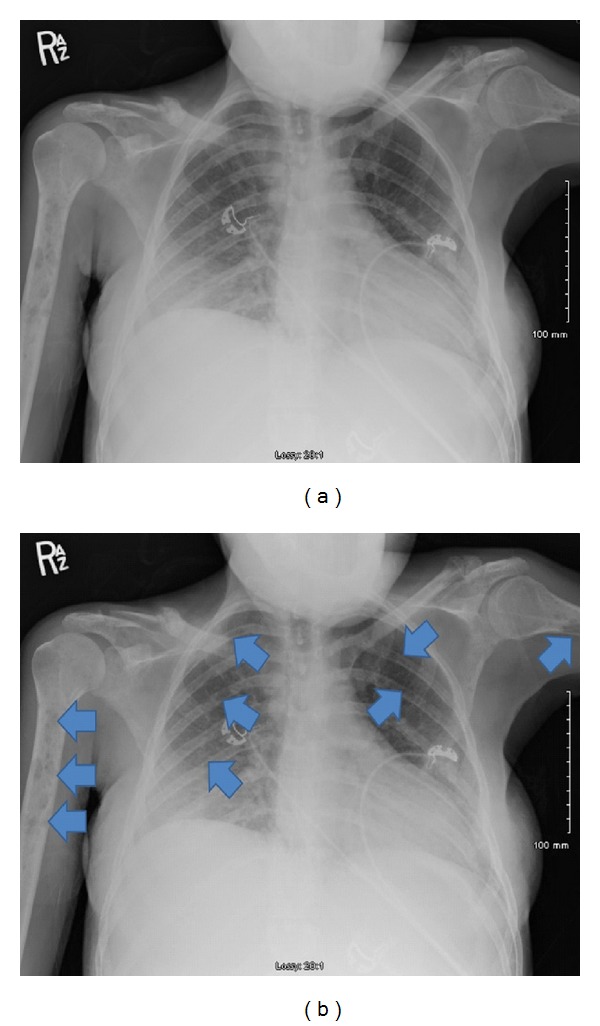
Chest radiograph of patient showing diffuse lytic bone lesions (indicated by blue arrows), most prominent in bilateral humeri and healed fracture deformities in the clavicles and ribs.

**Figure 2:**
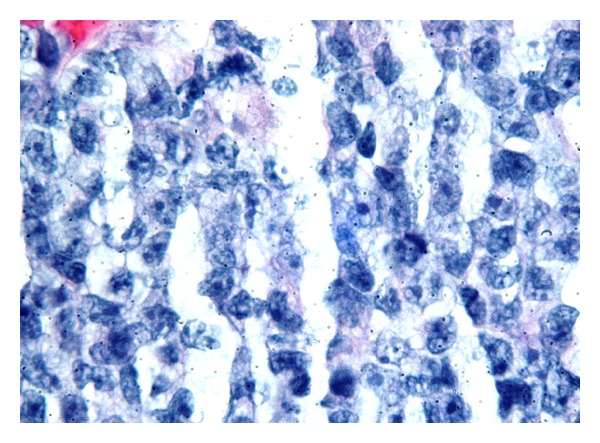
Minute fragment of core biopsy with morphology consistent with an intermediate to large cell aggressive T-cell lymphoma. These neoplastic cells were strongly positive for CD25 supporting the diagnosis of ATL.

**Figure 3:**
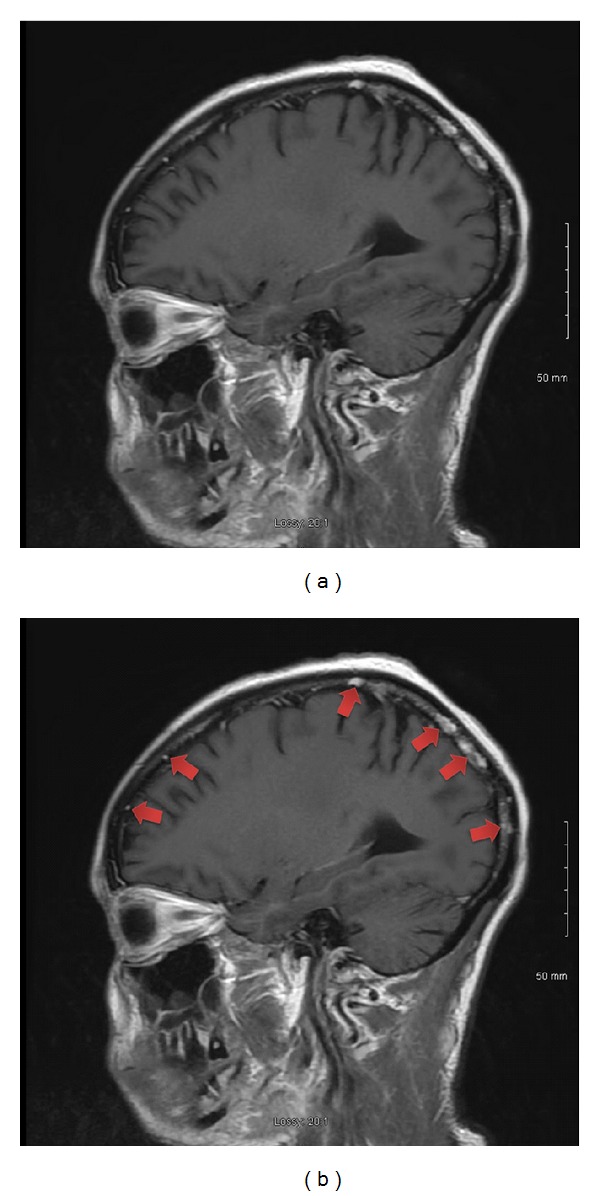
MRI of the brain revealed scattered subcortical T2-hyperintense foci and enhancing nodules in the calvarium (indicated by red arrows), consistent with metastatic disease.

A 27-year-old female Jamaican immigrant, at 28 weeks of age of gestation, presented with 1-week history of progressive confusion, poor appetite, vomiting, and joint pains. During admission, she was noted to have small nodular lesions around the mouth and neck, multiple bilateral tender cervical lymphadenopathy, multiple areas of tenderness on her right shoulder and ribs, and a gravid abdomen. Pertinent laboratory findings include leukocytosis (13,900/mL^3^), hypercalcemia (18.4 mg/dL), elevated ionized calcium (11.04 mg/dL), normal serum phosphorus (2.6 mg/dL), and elevated PTH-related protein (46 pg/mL) (see Table 1 for full discussion). HIV antibody test was negative. Chest radiograph revealed diffuse lytic lesions and fractures in the clavicles and ribs (Figure 1). Liberal hydration with intravenous normal saline was started and patient was given intravenous furosemide for hypercalcemia. Cautious emergent hemodialysis was instituted in the ICU to further control hypercalcemia. However, serum calcium remained elevated. Thus, intravenous calcitonin was started. Core biopsy of the largest cervical lymph node revealed intermediate to large peripheral T cells with immunophenotype classic for HTLV-1 lymphoproliferative disorder (Figure 2). Peripheral blood showed occasional atypical polylobulated T-lymphocytes. Qualitative HTLV-1 DNA test was positive. Screening test for HTLV-1 antibodies was also positive. This was subsequently confirmed by Qualitative HTLV-1 Line Immunoassay. Intravenous allopurinol was started because of subsequent hyperuricemia. In order to initiate chemotherapy promptly, it was decided through a multispecialty meeting to deliver the baby via cesarean section. Betamethasone was not given before delivery due to a high risk of tumor lysis. She gave birth to a baby girl, weighing 1280 grams, with an Apgar score of 1/2/3, who was admitted to the neonatal ICU. Following delivery, the patient received intravenous zoledronic acid and rasburicase for hypercalcemia and hyperuricemia, respectively. Fluoroscopy-guided lumbar puncture yielded cerebrospinal fluid that was positive for lymphoma cells. MRI of the brain revealed scattered subcortical T2-hyperintense foci and enhancing nodules in the calvarium, consistent with metastatic disease (Figure 3). Bone marrow biopsy and aspirate did not show evidence of involvement. The patient was started on E-CHOP (etoposide, cyclophosphamide, hydroxydaunorubicin, oncovin, and prednisone) chemotherapy. She also underwent Ommaya reservoir placement for intrathecal methotrexate. Her prolonged hospital course was complicated by deep venous thrombosis, central line-associated *Enterococcus faecalis* bacteremia, catheter-related fungal urinary tract infection, neutropenic fever, and central diabetes insipidus. These complications were treated accordingly and patient was discharged after 4 weeks of hospital stay. She will complete a total of six cycles of E-CHOP, as well as weekly intrathecal methotrexate. She will be referred for hematopoietic stem cell transplantation upon completion of chemotherapy.

## 3. Discussion

ATL, a highly aggressive T-cell non-Hodgkin lymphoma, is rarely encountered during pregnancy. Clinical features include hypercalcemia, lytic bone lesions, skin lesions, and lymphadenopathy. Infection with HTLV-1 is confirmed by antibody testing as well as viral DNA detection. Peripheral blood and bone marrow will show medium-sized lymphocytes with condensed chromatin and hyperlobulated nuclei known as “*flower cells.*” Lymph nodes have architectural effacement and large lymphoid cells. Flow cytometry shows T-cell associated antigens: CD2, CD4, CD3, CD5, and CD25 but usually lacks CD7. ATL can be manifested in four different clinical variants: acute, lymphoma-type, chronic, and smoldering. Patients with acute, lymphoma-type, and unfavorable chronic variants have poor prognosis and should be treated with combination therapy. The best chemotherapeutic regimen, however, is unclear. Our patient received E-CHOP, which is a standard regimen for peripheral T-cell lymphomas. Previous prospective trials have shown higher rates of response with the VCAP-AMP-VECP regimen (vincristine, cyclophosphamide, doxorubicin, prednisone, ranimustine, vindesine, etoposide, and carboplatin) [6]. However, some of these drugs are not available in the United States. Due to a high risk of central nervous system involvement, prophylactic intrathecal chemotherapy is also recommended. Although experience is limited, hematopoietic stem cell transplantation should be considered whenever possible. Further prospective trials are needed to validate the benefits of zidovudine and alpha interferon combinations to help achieve remission. Clinical trials are also underway for monoclonal antibodies against interleukin-2 receptor, CC chemokine receptor-4 (mogamulizumab), and CD25 (CAMPATH-1H), which are expressed in ATL cells [7]. The potential benefits of other agents including arsenic acid, pralatrexate, bendamustine, bortezomib, and lenalidomide are under investigation. There is no known effective treatment for recurrent and refractory ATL.

## 4. Conclusion

Treatment of ATL is challenging because of the lack of robust data on optimal chemotherapeutic regimens. More clinical trials are therefore needed to come up with definitive treatment guidelines. Whenever possible, patients with ATL should be encouraged to enroll in clinical trials. ATL is uncommonly seen in pregnancy, and it presents a dilemma with regard to prompt institution of chemotherapy to the mother without potentially harming the fetus. Delivery of the fetus when fetal lungs are mature followed by chemotherapy would be the best course of action. However, in cases where fetal lung maturity is not optimal, administration of intravenous steroids prior to delivery is not a reasonable option due to high risk for tumor lysis. A worse scenario is anticipated when ATL presents early in pregnancy when significant fetal prematurity precludes prompt delivery. Furthermore, maternal hypercalcemia and other concomitant metabolic derangements, as well as the HTLV-1 infection may have potential short-term and long-term adverse effects to the fetus.

## Figures and Tables

**Table 1:** Pertinent laboratory findings.

	Result	Normal
Calcium	18.4 mg/dL	(8.3–10.2)
Ionized calcium	11.4 mg/dL	(3.0–6.5)
Phosphate	2.6 mg/dL	(2.3–4.5)
Parathyroid hormone (PTH)	<2.5 pg/mL	(10–65)
PTH-related protein (PTH-rp)	46 pg/mL	(14–27)
Vitamin D Total 25(OH)	39 ng/mL	(30–100)
Creatinine	1.06 mg/dL	(0.6–1.2)
Uric acid	12.2 mg/dL	(2.6–8.1)
Angiotensin-converting enzyme (ACE)	54	(9–67)
Serum protein electrophoresis (SPEP)	Negative	
Urine protein electrophoresis (UPEP)	Negative	
Serum pH	7.38	(7.35–7.45)

Elevated calcium with normal phosphate and PTH levels points against a diagnosis of primary hyperparathyroidism. Normal vitamin D and ACE levels point against a diagnosis of sarcoidosis or granulomatous disease. Creatinine was normal. Uric acid was noted to be elevated. Negative SPEP and UPEP point against a diagnosis of multiple myelomas. A normal serum pH points against a diagnosis of milk-alkali syndrome. Elevated PTH-rp levels are highly suggestive of hypercalcemia of malignancy.
